# London Measure of Unplanned Pregnancy for South African women with mental illness: Exploring perspectives on pregnancy

**DOI:** 10.4102/sajpsychiatry.v24i0.1281

**Published:** 2018-10-05

**Authors:** Elsa du Toit, Esme Jordaan, Liezl Koen, Jukka M. Leppanen, Dana Niehaus

**Affiliations:** 1 Department of Psychiatry, Faculty of Health Sciences, Stellenbosch University, South Africa; 2 Panorama Healthcare Psychiatry Centre, South Africa; 3 Biostatistics Unit, Medical Research Council, South Africa; 4 Department of Statistics and Population Studies, University of the Western Cape, South Africa; 5 Stikland Hospital, South Africa; 6 Tampere Center for Child Health Research, School of Medicine, University of Tampere, Finland

## Abstract

**Introduction:**

Unplanned pregnancy is a community health concern. Research with South African women revealed the complexities surrounding pregnancy planning. Categorising pregnancies as either planned or unplanned is insufficient, as reducing a multidimensional concept to a dichotomous variable oversimplifies a complex matter.

**Methods:**

Pregnant females, 18 years and older with a primary DSM-IV-TR (APA 2000) diagnosis of psychiatric illness, are qualified for inclusion in this quantitative descriptive study. Participants completed a structured psychiatric assessment, including the London Measure of Unplanned Pregnancy (LMUP) during care as usual visits at two Maternal Mental Health Clinics.

**Results:**

Although 37.1% termed their pregnancy unplanned when asked dichotomously, the LMUP scores revealed that 50.6% of the 170 participants fell outside the ‘planned’ category. Worryingly, 73.3% of the women with unplanned or ambivalent pregnancies did not use contraception. Neither the women’s intention to fall pregnant nor their perception of the right timing for being pregnant could be predicted by the group (unplanned, ambivalent or planned) in which they fell; 82.6% of the unplanned group, 57.1% of the ambivalent group and 6.0% of the planned group indicated not wanting the baby. All the women in the ‘planned’ group agreed with their partner to have a baby. This holds true for 24.4% of the women in the other two groups.

**Conclusion:**

Results revealed similar findings as other studies in terms of contraception use, pregnancy timing, pregnancy intent, desire to have a baby, partner involvement and health-promoting behaviours during pregnancy. The large size of the ambivalent category emphasises that pregnancy planning cannot be viewed in terms of two dichotomous points, but should rather be thought of as a scale or continuum.

